# The gut-retina axis in diabetic retinopathy: a new paradigm for pathogenesis and therapeutic intervention

**DOI:** 10.3389/fimmu.2026.1868837

**Published:** 2026-08-25

**Authors:** Bainan Tong, Mingxuan Wang, Xinyue Yang, Lu Liu

**Affiliations:** Department of Ophthalmology, The Second Hospital of Jilin University, Changchun, China

**Keywords:** diabetic retinopathy, gut microbiota, gut-retina axis, microbial metabolites, probiotics

## Abstract

Diabetic retinopathy (DR) remains a leading cause of preventable blindness worldwide. However, current therapies predominantly target downstream vascular pathology and leave upstream drivers largely unaddressed. The emerging gut-retina axis paradigm reframes DR as a systemic disorder intricately linked to intestinal homeostasis. This review provides a comprehensive and updated synthesis of the gut-retina axis in DR, with three distinctive contributions that set it apart from earlier overviews. First, we systematically dissect the differential roles of specific microbial metabolites. We distinguish the opposing effects of primary versus secondary bile acids, the divergent bioactivities of conjugated versus unconjugated forms, and the dual inflammatory and protective functions of short-chain fatty acids. This analysis offers a nuanced mechanistic framework that moves beyond generalized descriptions of dysbiosis. Second, we critically evaluate the methodological heterogeneity that currently impedes cross-study comparability and propose practical standardization strategies to accelerate clinical translation. Third, we present an integrated overview of emerging therapeutic modalities, including next-generation probiotics, metabolite-targeted interventions, fecal microbiota transplantation, and natural product–based nanomedicines, while candidly addressing the translational gap between robust preclinical findings and limited human evidence. Importantly, we highlight how Mendelian randomization studies are beginning to establish causal links between specific gut taxa and DR risk, complementing traditional association-based reports. By integrating multi-omics perspectives, methodological rigor, and therapeutic innovation, this review offers a forward-looking roadmap for transforming gut-retina axis insights into actionable precision medicine strategies for DR.

## 1 Introduction

Diabetic retinopathy (DR) is one of the most common microvascular complications of diabetes and the leading cause of blindness among the working-age population worldwide. Its pathogenesis is complex and involves multiple interacting factors that damage retinal vascular and neural structures over time (1). Hyperglycemia is the primary driver of DR onset and progression. The core mechanisms triggered by chronic hyperglycemia include polyol pathway activation, accumulation of advanced glycation end products, upregulation of protein kinase C signaling, and metabolic disturbances in the hexosamine pathway. These processes damage the pericytes and endothelial cells of the retinal capillaries, disrupting the blood-retinal barrier (2). This disruption ultimately produces characteristic funduscopic microvascular changes: microaneurysms, hemorrhages, hard exudates, and cotton-wool spots. In advanced stages, it leads to retinal ischemia, pathological neovascularization, and fibroproliferation. Clinically, DR is traditionally classified into non-proliferative diabetic retinopathy (NPDR) and proliferative diabetic retinopathy (PDR). NPDR is characterized mainly by vascular leakage and occlusion, whereas PDR features pathological neovascularization accompanied by serious vision-threatening complications such as vitreous hemorrhage and tractional retinal detachment. Diabetic macular edema (DME), a common cause of vision loss, can occur at any stage of the disease.

In recent years, the research perspective has expanded considerably. Rather than focusing solely on ocular vascular changes, investigators now view DR as a local manifestation of a systemic disease closely tied to imbalances in metabolic, immune, and neuroendocrine networks. The introduction of the “gut–retina axis” concept has been particularly transformative. It has revealed intrinsic connections among intestinal microbiota ecology, systemic inflammation, and retinal pathology, thereby providing a novel framework for understanding DR pathogenesis and developing new prevention and treatment strategies (3, 4).

Currently, the main clinical approaches for managing DR include anti-vascular endothelial growth factor (VEGF) therapy, laser treatment, and vitrectomy. Each of these methods has significant limitations. Laser photocoagulation causes permanent retinal damage; vitrectomy is an invasive procedure; and although anti-VEGF therapy is considered the gold standard, it faces multiple challenges: non-response, secondary resistance, poor patient compliance due to the need for frequent repeated injections, and risks of injection-related endophthalmitis. Moreover, anti-VEGF therapy primarily targets existing neovascularization. It does not effectively address the upstream inflammation and neurovascular unit dysfunction that drive vascular pathology. This explains why some patients still experience persistent inner blood–retinal barrier leakage and retinal atrophy after treatment (5). Anti-inflammatory treatments such as corticosteroids serve as second-line options, but they carry side effects including elevated intraocular pressure and accelerated cataract formation.

Developing novel drugs that target the upstream pathological mechanisms of DR—such as systemic inflammation, oxidative stress, and mitochondrial dysfunction—has become a frontier in ophthalmic drug research. In recent years, various natural active compounds and small molecules with gut microbiota-modulating properties have shown potential in treating DR. These agents act through the gut microbiota–retina axis. By modulating gut microbiota composition and their metabolites (such as SCFAs and BAs), they indirectly suppress retinal inflammation and oxidative stress, offering a novel therapeutic avenue for DR (6, 7).

Despite growing interest in the gut–retina axis, existing reviews have notable limitations that constrain translational impact. Earlier overviews (3, 4) descriptively cataloged microbial compositional changes but lacked mechanistic dissection of individual metabolites, often treating LPS, TMAO, bile acids, and SCFAs as parallel mediators rather than interacting effectors with distinct, sometimes opposing, bioactivities. The comprehensive review by Jiang et al. (8) advanced mechanistic understanding yet did not critically examine the profound methodological heterogeneity—across sequencing platforms, confounder adjustments, and analytical pipelines—that undermines cross-study comparability. Nor did it systematically integrate emerging Mendelian randomization evidence or candidly assess the translational gap between robust preclinical findings and limited human data. To address these gaps, this review provides three distinctive contributions: (1) a mechanistic hierarchy of microbial metabolites emphasizing differential receptor signaling; (2) a critical methodological appraisal with standardization proposals; and (3) an integrated, clinically oriented evaluation of therapeutic modalities, offering a forward-looking roadmap for future research and translation.

Building upon this critical appraisal, the modern management strategy for DR is gradually shifting from traditional local ophthalmic treatments to a comprehensive intervention model that combines systemic metabolic regulation, anti-inflammation, and multi-organ protection. This review aims to systematically summarize the latest progress in this field, deeply analyze the molecular mechanisms of the gut–retina axis in DR, and evaluate the application prospects of various intervention strategies, with particular emphasis on the three distinctive contributions outlined above. We also discuss current research challenges and future directions, with the goal of providing a comprehensive reference for researchers in this emerging interdisciplinary field. To ensure methodological rigor and transparency, we systematically searched PubMed, Web of Science, Embase, and Scopus up to May 2026. We used a combination of keywords covering diabetic retinopathy, gut microbiota, microbial metabolites, and related interventions, with manual cross-referencing of eligible articles. The evidence synthesized through this approach forms the basis for our subsequent mechanistic, methodological, and therapeutic analyses.

### 1.1 The role of the gut-retina axis in diabetic retinopathy

The gut-retina axis is a cross-organ communication framework derived from the concept of the “gut-brain axis”. It refers to the bidirectional signaling pathways through which the gut and the retina influence each other. According to this framework, the gut microbiota and its metabolites, gut barrier function, the gut immune system, and neuroendocrine signals can collectively influence retinal homeostasis and pathological processes (8). The gut microbiota is a complex ecosystem comprising trillions of microorganisms, including bacteria, fungi, and viruses. Its composition is dynamically shaped by host and environmental factors. A healthy gut microbiota is crucial for maintaining host homeostasis, including nutrient processing, immune response regulation, and intestinal wall protection. Dysbiosis of the gut microbiota may lead to systemic inflammation and subsequently trigger various diseases, including DR (9, 10).

**Figure 1:**
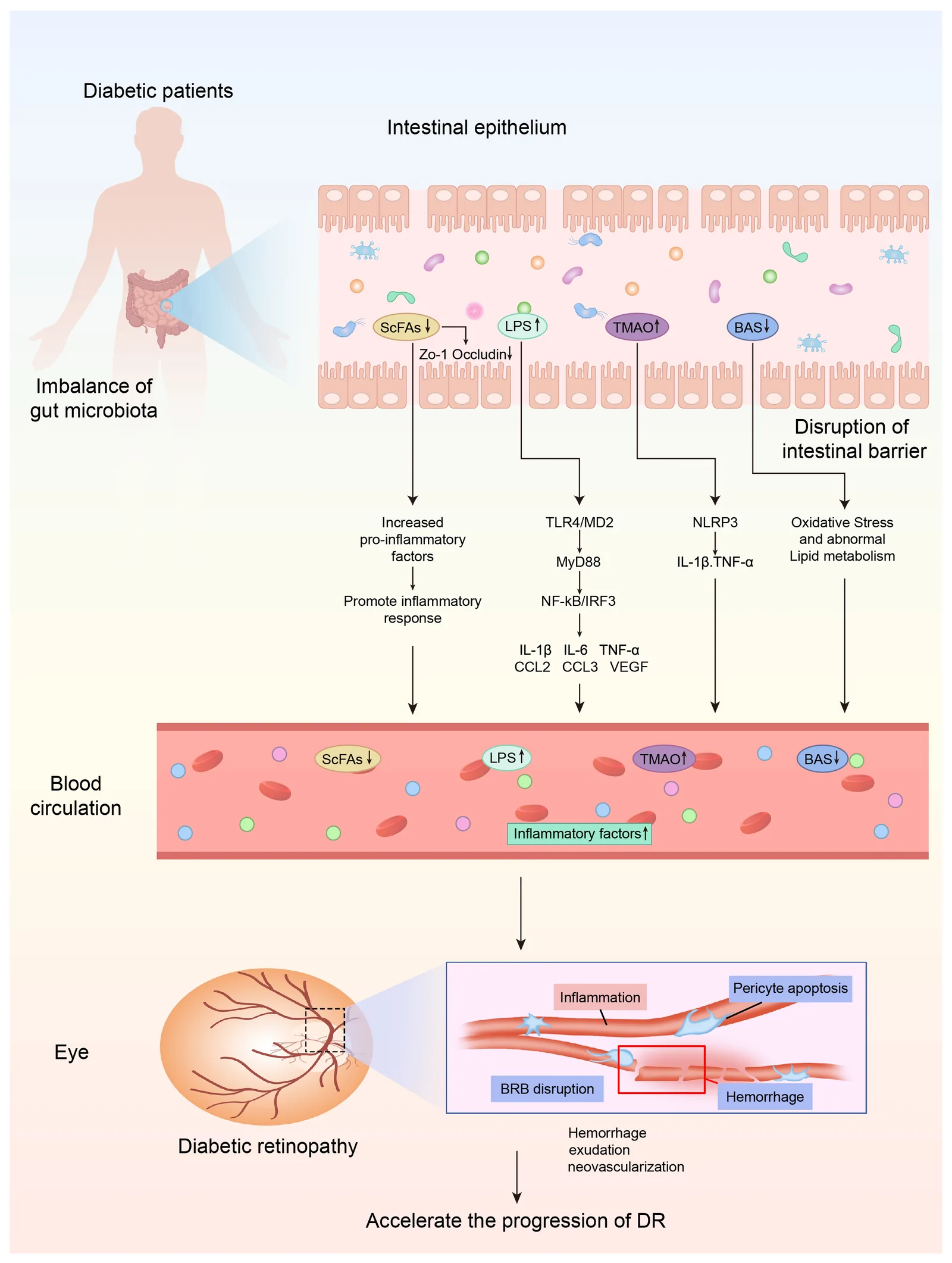
This diagram illustrates how gut microbiota dysbiosis drives diabetic retinopathy (DR) progression. Gut barrier disruption increases circulating LPS and TMAO while reducing SCFAs and BAs. These metabolites activate TLR4/MyD88/NF-kB signaling and NLRP3 inflammasome, releasing inflammatory cytokines (IL-1β, IL-6, TNF-α), chemokines (CCL2, CCL3), and VEGF. This systemic inflammation disrupts the blood-retinal barrier, causing pericyte apoptosis, hemorrhage, exudation, and neovascularization—ultimately accelerating DR. The gut-retina axis represents a promising therapeutic target.

In patients with DR, alterations in the intestinal microbiota create a state of dysbiosis that serves as a key driver of disease progression (**Figure 1**). This dysbiosis has several downstream consequences that directly impact the retina. Firstly, it promotes the breakdown of the intestinal barrier, allowing bacterial components such as lipopolysaccharide (LPS) to enter the circulatory system. This translocation triggers systemic and retinal inflammation—a hallmark of DR pathogenesis (11). Second, dysbiosis may increase the production of reactive oxygen species (ROS) by influencing host metabolism, thereby inducing oxidative stress in the retina. Oxidative stress is a core pathogenic mechanism that can damage cellular components, induce apoptosis, and promote vascular lesions (12). Third, through systemic inflammation and metabolic disturbances, dysbiosis indirectly influences pro-angiogenic factors such as VEGF, thereby promoting the abnormal angiogenesis that characterizes late-stage DR (13). Finally, neurotoxic metabolic products and chronic inflammation arising from flora imbalance may directly damage retinal neurons and glial cells, accelerating the early neurodegenerative process in DR (4, 11).

## 2 Mechanisms of the gut microbiota in diabetic retinopathy

### 2.1 Characteristic alterations in gut microbiota composition

The gut microbiota is a vast community of thousands of microbial species. Their genes and metabolic products are essential for multiple host metabolic pathways and for maintaining overall homeostasis. When the gut microbiota becomes imbalanced, systemic inflammatory responses can ensue, leading to various diseases including DR (14).

Although findings are not universally consistent across all studies, several reports indicate trends in microbial richness and compositional shifts in DR patients. For example, Bai et al. found that changes in the microbial composition of DR patients were associated with dysregulation at the phylum level, involving Firmicutes, Bacteroidetes, Synergistota, and Desulfobacterota. At the genus level, the DR group showed increased abundance of Bacteroides, Megamonas, *Ruminococcus torques* group, Lachnoclostridium, and Alistipes. Conversely, levels of Blautia, Eubacterium hallii subgroup, Collinsella, Dorea, Romboutsia, Anaerostipes, and Fusicatenibacter were decreased (15). A Mendelian randomization study identified Christensenellaceae and Peptococcaceae as protective factors against DR. In contrast, Ruminococcaceae UCG_011, the Enterococcus rectal subgroup, and Adlerkreutzella were associated with increased DR risk. At the phylum, class and order levels, no intestinal microbial classifications were found to have a causal relationship with DR (16).

Other research has revealed unique microbial β-diversity differences among DR patients, T2DM patients, and healthy controls. Key features include alterations in the Firmicutes/Bacteroidetes ratio, a reduction in butyrate-producing bacteria, and an increase in LPS-expressing and pro-inflammatory bacteria within the Bacteroidetes and Proteobacteria phyla. At the genus level, DR patients exhibit a more complex pattern of microbiota changes. Compared with healthy individuals, DR patients have an enrichment of Faecalibacterium, Roseburia, Lachnospira and Romboutsia, while Akkermansia is reduced. Compared with diabetes patients without retinopathy, DR patients have an enrichment of five genera including Prevotella, while Bacillus, Veillonella and Pantoea are reduced. These changes reflect the dual characteristics of the intestinal microbiota in DR patients: they retain the microbiota dysbiosis features associated with diabetes, while also having DR-specific alterations (17).

Overall, the evidence indicates that alterations in the gut microbiota are associated with DR and its severity. These alterations may exert their effects through multiple mechanisms, influencing vascular permeability, vascular cell adhesion molecule-1 (VCAM-1), hypoxia-inducible factor-1 (HIF-1), B cells, and insulin levels. Modulating gut microbiota composition may thus represent a novel strategy for preventing DR (18).

### 2.2 Mechanisms of action of key microbial metabolites

The metabolites generated by the gut microbiota serve as an important bridge connecting the intestine and the retina. These metabolic products are numerous and functionally diverse, and they play a key role in the occurrence and development of DR (8). The gut microbiota can produce metabolic products such as LPS, TMAO, BAs, and SCFAs. These metabolites are absorbed by the colon and released into the bloodstream via the portal vein and are then circulate systemically and eventually reach the retina. This systemic circulation process is accompanied by regulatory alterations in cytokines, proteins, and other substances (19). For instance, these microbial products can promote or inhibit the production of inflammatory factors and activate immune regulation, thereby inducing a chronic, persistent low-grade inflammatory response that contributes to DR progression (3).

#### 2.2.1 Lipopolysaccharide

Elevated LPS levels in DR patients are likely related to gut dysbiosis and intestinal barrier damage (17). Under hyperglycemic conditions, the intestinal microecology becomes unbalanced, with an increase in Gram-negative bacteria (such as certain Bacteroides, Proteobacteria, Escherichia, and Enterobacter). Hyperglycemia and its metabolites also downregulate tight junction proteins in intestinal epithelial cells, increasing permeability and creating a “leaky gut.” LPS then enters the portal venous circulation through this damaged barrier (20). Once in the systemic circulation, LPS acts as a potent inflammatory driver, triggering and exacerbating chronic low-grade inflammation both systemically and within the retina. It activates various retinal cells via its receptor, Toll-like receptor 4 (TLR4), leading to the upregulation of pro-inflammatory cytokines, chemokines, and pro-angiogenic factors (21).

**Figure 2:**
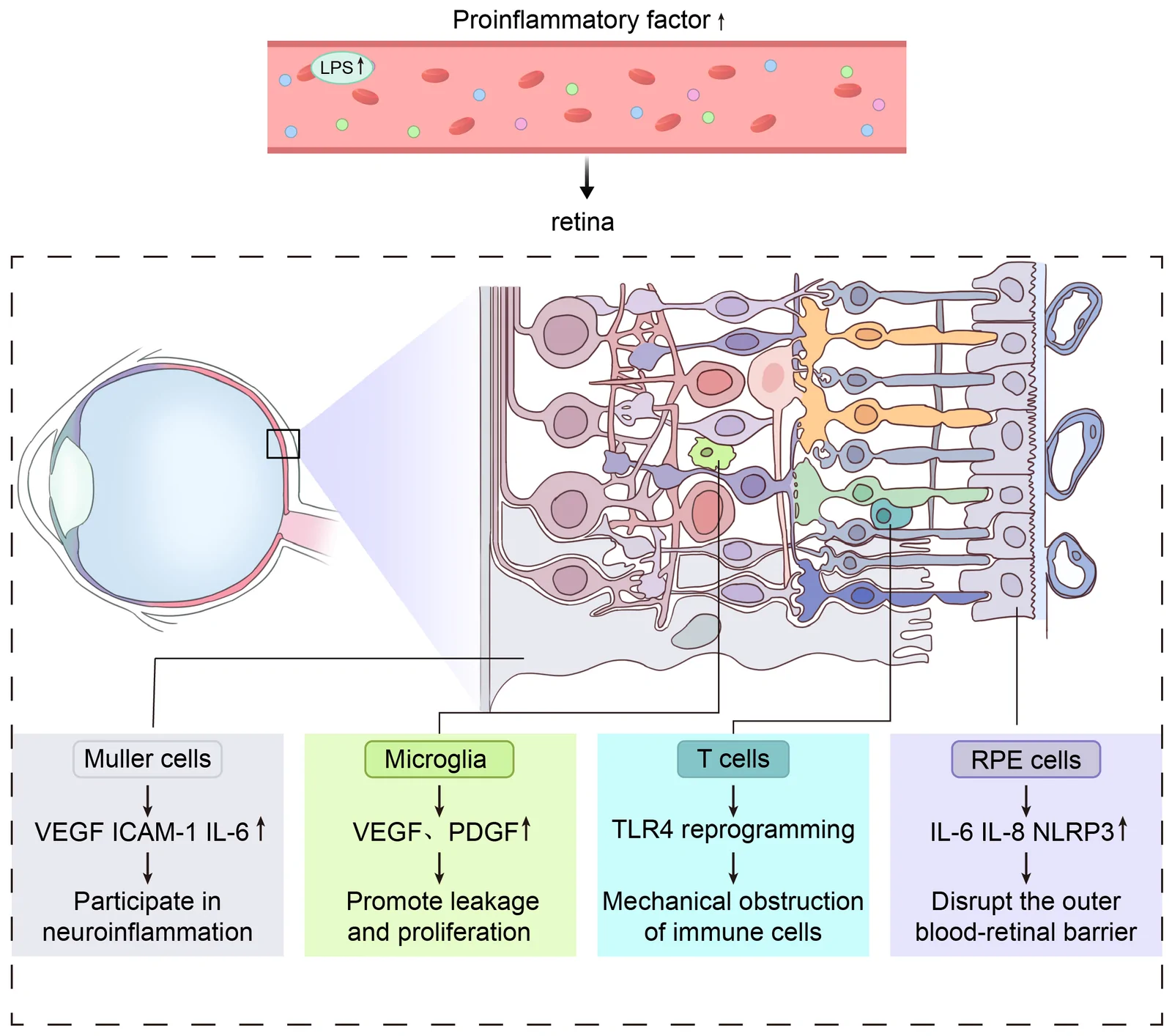
This diagram illustrates how elevated pro-inflammatory factors and LPS drive diabetic retinopathy (DR) through multicellular retinal responses. LPS activates Müller cells to release VEGF/ICAM-1/IL-6 promoting neuroinflammation; Microglia upregulate VEGF/PDGF promoting vascular leakage. T cells undergo TLR4 reprogramming causing immune cell mechanical obstruction; RPE cells secrete IL-6/IL-8/NLRP3 disrupting the outer blood-retinal barrier. These coordinated cellular events collectively disrupt retinal homeostasis and accelerate DR progression.

LPS exerts specific damaging effects on retinal cells through the following mechanisms:

1. Activation of retinal microglia: Prolonged LPS stimulation drives microglia toward excessive polarization to the pro-inflammatory M1 phenotype (22). Activated microglia show enhanced proliferation, migration, and phagocytic capacity, and release large amounts of inflammatory factors. By secreting VEGF and PDGF, they promote angiogenesis and leakage. Through phagocytosis of retinal endothelial cells, they directly lead to the formation of acellular capillaries. Pericytes co-cultured with activated microglia have enhanced ability to produce ROS and pro-inflammatory mediators, which damages the BRB (23).
2. Driving reactive gliosis: LPS upregulates the expression of inflammatory mediators such as VEGF, ICAM-1, and IL-6 in Müller cells through TLR4 signaling. Activated Müller cells may play a role as non-classical antigen-presenting cells and participate in neuroinflammation. LPS also disrupts the potassium channel localization and water-potassium transport in Müller cells, leading to retinal edema and neurovascular unit dysfunction (24).
3. Damage to the blood-retinal barrier: LPS converts RPE cells into a pro-inflammatory state, causing them to secrete factors such as IL-6 and IL-8. Through autocrine loops, these factors promote RPE degeneration and disrupt tight junctions (25). Simultaneously, LPS induces endoplasmic reticulum stress and excessive activation of the NLRP3 inflammasome. Chronic exposure results in persistent inflammation that impairs the barrier and phagocytic functions of RPE (26). LPS renders retinal endothelial cells (RECs) extremely sensitive to inflammatory factors. It upregulates chemokines and adhesion molecules (such as ICAM-1, VCAM-1) and causes leukocyte stasis. Leukocyte adhesion leads to endothelial swelling and capillary occlusion. LPS also induces endoplasmic reticulum stress and apoptosis in RECs (27).
4. Recruiting peripheral immune cells exacerbates ischemia and hypoxia: Persistent inflammatory signals in the retina recruit peripheral macrophages, T cells and others through the leaking vascular walls into the retina. These infiltrated macrophages are reprogrammed via the TLR4 pathway to perform pro-inflammatory and pro-angiogenic functions similar to those of microglia. The accumulation of immune cells inside and outside blood vessels mechanically blocks vessels, increases non-perfusion areas, aggravates retinal ischemia and hypoxia, and ultimately drives pathological neovascularization (28) (**Figure 2**).

#### 2.2.2 Trimethylamine N-oxide

TMAO is a metabolite produced by the gut microbiota from dietary nutrients such as choline and carnitine and is further oxidized in the liver. Liu et al. demonstrated that in T2DM patients, elevated plasma TMAO levels were independently associated with a higher risk of DR and more severe lesions (29). In the differential diagnosis of diabetic and non-diabetic retinopathy, high TMAO levels are associated with DR but not with non-DR. Elevated TMAO may be a risk factor for DR development and progression, as well as for other diabetic complications (30). Several mechanisms may explain how TMAO contributes to DR: (1) TMAO may exacerbate the metabolic abnormalities of diabetes by enhancing insulin resistance and altering liver cholesterol metabolism, thereby promoting DR (31). (2) TMAO can induce increases in pro-inflammatory cytokines such as TNF-α and IL-1β. Inflammation is a core mechanism in DR progression (32). (3) TMAO can directly activate the inflammatory signaling pathways (such as NF-κB) in vascular endothelial cells, promote oxidative stress, accelerate endothelial cell senescence and dysfunction, and increase vascular permeability. These effects are consistent with the pathological features of DR (33). (4) Diabetes is often accompanied by gut microbiota imbalance, which may alter TMAO production. Changes in microbial composition (such as alterations in the ratio of Firmicutes to Bacteroidetes) are also associated with DR risk and TMAO levels.

Xue et al. conducted more detailed mechanistic studies, showing that in DR, gut microbial products (LPS, TMAO, etc.) activate the P2X7/NLRP3 inflammasome in retinal endothelial cells, triggering an inflammatory storm, apoptosis and pyroptosis (34). Another study has systematically linked the TMAO molecular network with DR for the first time. It is proposed that TMAO may drive inflammation, oxidative stress and apoptosis by regulating core targets such as CASP3/CXCR4/MAPK1, thereby participating in PDR progression. This provides a theoretical framework for understanding the gut–retina axis and for developing TMAO-based diagnostic and therapeutic strategies (35).

#### 2.2.3 Bile acids

Bile acids are endogenous molecules synthesized in the liver from cholesterol. They exist in bile as ionic salts. Bile acids and their receptors—particularly TGR5 and FXR—have emerged as important regulatory factors in metabolic diseases (8). TGR5 is a G protein-coupled receptor that is widely distributed in various tissues, including the retina. Studies have shown that TGR5 receptor activation can alleviate diabetic retinopathy by inhibiting the RhoA/ROCK signaling pathway. The RhoA/ROCK signaling pathway plays a role in vascular contraction, cytoskeletal rearrangement, and inflammatory responses, and its abnormal activation is associated with microvascular damage and neuroinflammation in DR (36). Serum BA levels are associated with DR risk and progression. A study of T2DM patients found that changes in serum bile acid and unsaturated fatty acid profiles correlated with DR risk (37).

Differential mechanisms of action between primary and secondary bile acids:Primary BAs are synthesized in the liver and mainly include cholic acid (CA) and chenodeoxycholic acid (CDCA). Secondary bile acids such as deoxycholic acid (DCA) and lithocholic acid (LCA) are produced by gut microbiota through the biotransformation of primary BAs (38). These two classes of bile acids act through markedly different mechanisms in DR. Primary bile acids, especially CDCA, are high-affinity agonists of the nuclear receptor FXR. FXR activation usually inhibits *de novo* synthesis of bile acids and regulates glucose metabolism. However, in the pathological state of DR, persistent abnormal FXR signaling may lead to increased retinal vascular permeability. In contrast, secondary bile acids such as LCA and DCA are potent agonists of the membrane receptor TGR5 (39). TGR5 activation can alleviate retinal microvascular endothelial dysfunction by inhibiting the RhoA/ROCK pathway, thereby exerting protective effects. In addition, TGR5 can block the cGAS/STING inflammatory pathway by inhibiting GRP75-mediated endoplasmic reticulum–mitochondria coupling, thus improving Müller cell homeostasis (40).

Bioavailability and effects of conjugated and unconjugated bile acids: In the liver, BAs conjugate with glycine or taurine to form conjugated bile acids. This conjugation significantly lowers their pKa and increases water solubility. Conjugated bile acids such as glycocholic acid (GCA), glycodeoxycholic acid (GDCA), and glycochenodeoxycholic acid (GUDCA) exhibit unique advantages in protecting retinal pigment epithelium (RPE) tight junctions from oxidative stress damage (41). *In vitro* studies show that glycine-conjugated bile acids effectively inhibit VEGF-induced angiogenesis in choroidal endothelial cells, suggesting potential therapeutic value in suppressing pathological neovascularization in advanced DR. In contrast, unconjugated BAs, due to their higher lipid solubility, can more easily penetrate cell membranes but are also more cytotoxic. The gut microbiota uses bile salt hydrolase (BSH) to deconjugate conjugated bile acids into unconjugated forms, which are then converted into secondary BAs. This process alters the overall signaling properties of the bile acid pool (42). Therefore, gut microbiota-mediated deconjugation not only affects the efficiency of enterohepatic circulation but also indirectly regulates retinal inflammation and metabolic status by modifying local BA composition.

The gut microbiota and its metabolic products have significant impacts on host metabolism and inflammation. BAs are key regulatory molecules in the gut microbiota–host interaction and play a central role in this bidirectional crosstalk (3, 43). One study demonstrated that a synbiotic preparation of probiotics and β-glucan from highland barley can improve diabetic retinopathy in db/db mice by regulating the gut-retina axis. Bile acids may act as key signaling molecules in this process (43). DR development is closely linked to abnormal lipid metabolism and oxidative stress. As lipid metabolites, BAs and their metabolites may directly or indirectly affect lipid homeostasis and redox status within the retina. Elevated levels of homocysteine are common in diabetic patients and are associated with the risk of DR and mitochondrial damage. Changes in BAs metabolism may interact with abnormal homocysteine levels, further influencing DR pathophysiology (44).

Although evidence suggests a connection between BAs and DR, the specific molecular mechanisms and therapeutic potential require further investigation. Future research should focus on several areas: clarifying the specific roles of bile acid receptors such as TGR5 and FXR in retinal cells under diabetic conditions; comprehensively profiling changes in different bile acid species across DR stages using metabolomics; and exploring how gut microbiota modulation of bile acid metabolism affects retinal health. Future therapeutic strategies could involve precisely modulating specific bile acid subtypes—for example, by regulating gut microbiota composition with probiotics or prebiotics to enhance production of beneficial secondary bile acids, or by developing highly selective TGR5 agonists for multi-target DR intervention. Moreover, combining metabolomics to monitor bile acid profiles in patients’ serum and intraocular fluid may provide new biomarkers for early DR diagnosis and personalized treatment.

#### 2.2.4 Short-chain fatty acids

SCFAs are mainly produced by gut microbial fermentation of polysaccharides and other carbohydrates. The gut microbiota and its metabolites, SCFAs (mainly acetate, propionate, and butyrate), have a bidirectional relationship with retinal health through immune regulation, metabolic influence, and possible direct signaling pathways (45). In diabetic patients, plasma SCFA levels are significantly lower in those with DR than in those without DR. Qin et al. found that in a diabetic mouse model, dietary acetate supplementation reduced total retinal layer thinning and vascular loss, independent of changes in body weight or blood glucose. It also significantly reduced infiltration of CD45^+^CD11b^+^ myeloid cells into the retina—particularly the F4/80^+^CD206^+^CD11c^-^ population—indicating suppression of excessive retinal immune responses (46).

Butyrate, a key SCFA, has shown potential anti-diabetic effects. Sodium butyrate alleviated the thinning of the total retinal thickness, inner layer and middle layer caused by diabetes. It inhibits the activation of retinal microglia. Sodium butyrate significantly increased the expression of tight junction proteins (ZO-1 and Occludin) in the small intestine of diabetic mice, indicating that it helps to repair the damaged intestinal barrier. Butyrate also reshaped the gut microbiota composition in ways that promoted SCFA metabolism (47).

SCFAs may influence DR through the following mechanisms:(1) Chronic low-grade inflammation caused by diabetes is a key factor in DR progression (48). SCFAs, particularly butyrate, can suppress inflammatory responses and reduce the production of pro-inflammatory cytokines, thereby potentially alleviating retinal inflammation and protecting the retina from damage (47). (2) SCFAs have been proven to improve insulin sensitivity. In a gestational diabetes mouse model, gut dysbiosis led to reduced SCFAs, which in turn caused adipose tissue macrophage polarization and insulin resistance (49). Insulin resistance is a characteristic manifestation of T2DM and also a risk factor for DR. By improving insulin action, SCFAs may indirectly slow DR progression. (3) Diabetic patients often have increased intestinal permeability, allowing bacterial products such as LPS to enter the bloodstream and trigger systemic inflammation (50). SCFAs may reduce DR risk by strengthening tight junctions, preserving intestinal barrier integrity, and limiting absorption of pro-inflammatory substances. (4) Although the specific mechanisms require further study, SCFAs appear to protect retinal vessels through their effects on endothelial cells and oxidative stress (45).

### 2.3 Regulatory mechanisms of molecular signaling pathways

**Figure 3:**
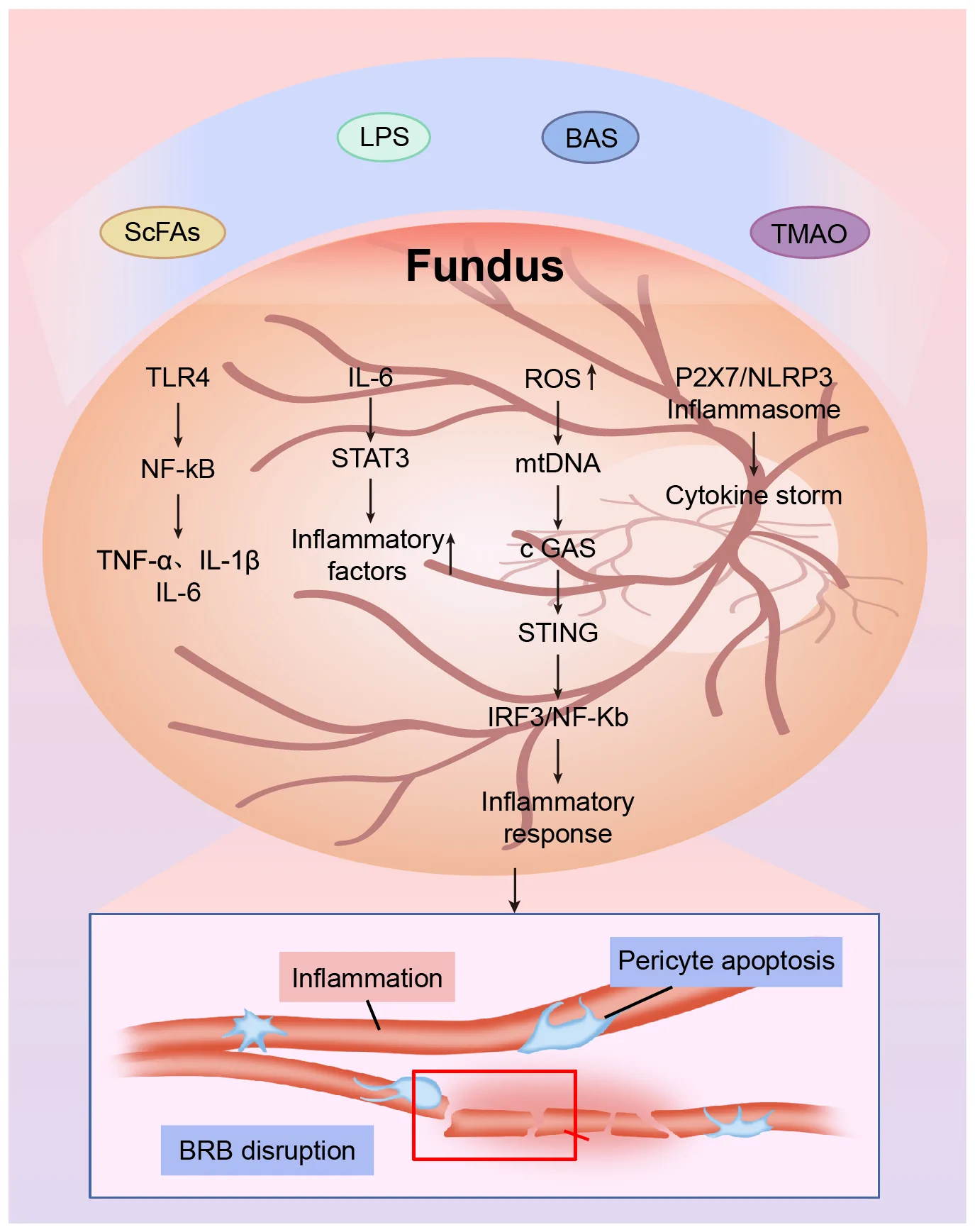
This diagram illustrates the signaling pathways through which gut-derived metabolites drive retinal inflammation and diabetic retinopathy (DR) progression. Circulating LPS and TMAO, along with reduced SCFAs and BAs, activate multiple inflammatory cascades in the retina. Key pathways include: TLR4-mediated inflammatory factors production; IL-6/STAT3 signaling; cGAS-STING pathway activation by mitochondrial DNA, leading to IRF3/NF-κB-driven inflammatory responses; and P2X7/NLRP3 inflammasome activation. These converging pathways trigger a cytokine storm (TNF-α, IL-1β, IL-6), culminating in pericyte apoptosis, inflammation, and blood-retinal barrier (BRB) disruption—key pathological events accelerating DR. This diagram highlights the gut-retina axis and its downstream inflammatory signaling networks as central to DR pathogenesis.

Gut dysbiosis contributes substantially to DR development, and the signaling network of the gut–retina axis is central to understanding this connection. The gut-retina axis affects DR through multiple molecular signaling pathways, primarily involving inflammation, oxidative stress, neurovascular dysfunction, and mitochondrial impairment (**Figure 3**).

#### 2.3.1 Inflammatory pathways

TLR4/NF-κB pathway: Increased intestinal permeability allows bacterial products such as LPS to enter the bloodstream, where they activate Toll-like receptor 4 (TLR4) in the retina. TLR4 is a key innate immune receptor that recognizes pathogen-associated molecular patterns (PAMPs) and damage-associated molecular patterns (DAMPs), thereby activating the downstream NF-κB signaling pathway (51). NF-κB is a major transcription factor that regulates expression of various pro-inflammatory genes, including TNF-α, IL-1β, and IL-6. In DR patients, hyperglycemia-induced oxidative stress and inflammation are central to pathogenesis, and the overactivation of the TLR4/NF-κB pathway is considered a key driver of retinal inflammation, oxidative stress, increased vascular permeability, and retinal damage (8, 52).

In a gestational diabetes mouse model, gut dysbiosis reduced SCFAs and promoted adipose tissue macrophage polarization, increasing LPS production and inducing inflammatory factors via the TLR4/NF-κB pathway (49). In high glucose-treated human retinal microvascular endothelial cells (HRMECs), the activation of the TLR4/NF-κB pathway leads to intensified oxidative stress and inflammatory responses. Albiflorin alleviated high glucose–induced oxidative stress and inflammation in HRMECs by inhibiting this pathway (53). Retinal neurodegeneration occurs early in DR, and studies suggest it may be related to TLR4/NF-κB–mediated neuroinflammation. Asiatic acid alleviates the pathological process of streptozotocin (STZ)-induced DR in rats by regulating the polarization of microglia mediated by the TLR4/MyD88/NF-κB p65 pathway (54).

IL-6/STAT3 pathway: Gut dysbiosis leads to damage of the intestinal barrier, allowing bacterial products to translocate into the bloodstream. These circulating bacterial products activate systemic inflammation and elevate pro-inflammatory cytokines such as IL-6. Elevated IL-6 activates the STAT3 pathway in the retina, promoting an inflammatory cascade and exacerbating DR lesions (55). Abnormal IL-6/STAT3 activation intensifies the retinal inflammatory microenvironment and contributes to vascular pathology by regulating factors such as VEGF (56). The gut microbiota influences the systemic inflammatory state by modulating the host immune system. Dysregulated microbiota may cause abnormal immune cell activation and excessive IL-6 production, which in turn affects retinal health (55). Gut microbiota imbalance may also indirectly affect the IL-6/STAT3 pathway through other mechanisms—such as oxidative stress and endoplasmic reticulum stress—thereby influencing DR.

#### 2.3.2 Oxidative stress pathway

ROS are normal by-products of cellular metabolism. However, when physiological homeostasis is disrupted, elevated ROS levels cause oxidative stress, leading to cellular damage, inflammatory responses, and various chronic diseases (57). Bacterial products trigger systemic low-grade inflammation and oxidative stress. Through the bloodstream, they reach the retina, damage the blood-retinal barrier, and directly or indirectly intensify retinal oxidative stress and inflammation. Studies have shown that the STING pathway is a widely expressed adaptor protein. When activated by the cytoplasmic DNA sensor cGAS, it induces IRF3 and NF-κB activation, thereby causing inflammation. This inflammatory response may be an important driver of oxidative stress in the gut-retina axis (58). SCFAs produced by the gut microbiota regulate host energy metabolism, immune responses, and oxidative stress levels. Dysbiosis reduces SCFA production, weakening their anti-inflammatory and antioxidant effects and making the retina more vulnerable to oxidative damage (45). Some compounds have been proven to provide neuroprotective effects via the gut–retina axis. Oral administration of 7,8-dihydroxyflavone (7,8-DHF) protects retinal ganglion cells by regulating the gut-retina axis and inhibiting ferroptosis, especially through the IDA-AhR-ALDH1A3-FSP1 pathway. 7,8-DHF also altered the gut microbiota, increasing the Parasutterella genus, which was associated with higher levels of IDA (59).

#### 2.3.3 Metabolic pathways

The polyol pathway, hexosamine pathway, and advanced glycation end products/advanced glycation end products receptor (AGE/RAGE) pathway play key roles in DR development and exert their effects through the gut–retina axis. AGEs directly damage intestinal epithelial cells, impairing barrier function and increasing permeability (8). The intestinal microbial components and metabolites that enter the circulation activate systemic immune cells, triggering inflammatory responses. This systemic inflammation—including RAGE-mediated inflammation—may ultimately affect retinal health. Shi et al. demonstrated that long-term intake of β-lactoglobulin-bound AGEs induced colonic inflammation in mice by regulating the RAGE (TLR4)/MYD88/NF-κB signaling pathway and the gut microbiota (60).

The STING pathway is an underappreciated in the gut-retina axis. After activation by the cytosolic DNA sensor cGAS, it induces IRF3 and NF-κB activation, leading to inflammation. Gut microbiota dysbiosis influences retinal inflammation through the STING pathway (58). Metabolic pathways damage retinal microvasculature and neural structures by inducing oxidative stress, inflammation, and cellular dysfunction. They also affect intestinal barrier integrity and microbiota homeostasis, indirectly participating in gut–retina axis regulation. Targeting these metabolic pathways and the gut-retinal axis offers new therapeutic strategies for DR. SGLT2 inhibitors have improved outcomes in diabetic nephropathy, suggesting potential benefits for diabetic complications including DR (61).

#### 2.3.4 Other relevant pathways

PKC isozymes are the main regulators of inflammatory signal transduction. Hyperglycemia-induced PKC activation contributes to retinal vascular injury and inflammation, representing a key mechanism of DR (62). Pomegranate peel extract (PPE) alleviated DR by inhibiting the PI3K/AKT/HIF-1α/VEGF pathway and modulating the gut microbiota. PPE also improved gut dysbiosis in DR rats, notably by increasing Akkermansia muciniphila, which alleviates inflammation and oxidative stress (63). Dysregulation of neurotrophic factors and the gut–retina axis interact to significantly affect retinal health and disease. Gut dysbiosis can cause systemic inflammation and oxidative stress, key drivers of retinal neurodegeneration. Neurotrophic factors have anti-inflammatory and antioxidant properties, and reduced levels may render the retina more susceptible to inflammatory and oxidative damage (64). Retinal cells, especially photoreceptors, retinal pigment epithelium (RPE), and retinal ganglion cells (RGC), contain abundant mitochondria and are thus highly sensitive to mitochondrial dysfunction. Under hyperglycemic conditions, mitochondrial dysfunction in retinal neurons, especially increased DRP1-dependent mitochondrial fission, is a key factor driving photoreceptor abnormalities (65).

### 2.4 Inflammation and immune regulation mechanisms

DR development is closely linked to chronic low-grade inflammation. Hyperglycemia increases oxidative stress, which in turn activates inflammatory pathways, promotes retinal vascular dysfunction, and increases capillary permeability and vascular leakage. Gut dysbiosis promotes inflammatory responses in DR through multiple mechanisms (8). Intestinal barrier dysfunction is frequently observed in diabetic patients and DR animal models, making it easier for gut microorganisms and their metabolites to enter the systemic circulation. After entering the circulation, LPS and other microbial components activate immune cells in the intestine and retina. In the retina, microglia— resident immune cells of the retina—are activated and release pro-inflammatory cytokines such as IL-1β, IL-6, TNF-α and MCP-1, further exacerbating retinal inflammation and vascular dysfunction (66). Peptidoglycan (PGN) and other gut microbial components can translocate to the circulation, bind to Toll-like receptor 2 (TLR2) in the retina, increase blood–retinal barrier permeability, and promote DR (67).

The gut microbiota not only directly drives inflammation but also indirectly regulates the immunopathological process of DR by influencing the balance of systemic immune cells and metabolic products. In DR, the balance between helper T cell 17 (Th17) and regulatory T cells (Treg) is disrupted. Th17 cells mainly produce pro-inflammatory cytokines such as IL-17A, promoting inflammation and tissue damage. In contrast, Treg cells suppress inflammatory responses by secreting cytokines like IL-10 and TGF-β, maintaining immune homeostasis. Under diabetic conditions, gut dysbiosis may impair dendritic cell function and alter metabolic products, promoting Th17 cell differentiation while inhibiting Treg cell function, thereby exacerbating retinal inflammation (68).

## 3 Technical methods for studying the gut-retina axis in diabetic retinopathy

### 3.1 Analysis of gut microbiota composition and diversity

16S rRNA gene sequencing is the most commonly used method. By amplifying and sequencing specific variable regions of the bacterial 16S rRNA gene, researchers can classify and identify intestinal microbial communities to the genus and even species level. A study conducted 16S rRNA gene sequencing on fecal samples from 45 PDR patients and 90 diabetic patients without retinopathy (NDR). The results showed reduced microbial diversity in PDR patients and altered abundance of certain bacteria, including a shift in the Firmicutes/Bacteroidetes ratio (69). Metagenomic shotgun sequencing differs from 16S rRNA gene sequencing in several respects. This approach identifies microbial species and provides functional information about the microbiome, including gene content and metabolic pathways. Using metagenomic sequencing combined with untargeted metabolomics, studies have revealed significant changes in both the composition and function of the gut microbiota in DR patients. Specific DR-associated species have been identified, including Eubacterium hallii, Firmicutes bacterium, and Alistipes finegoldii (70). These techniques help researchers identify specific gut microbiota changes in DR patients, providing a basis for subsequent mechanistic studies and therapeutic target screening.

16S rRNA gene sequencing has become the preferred tool for exploring gut microbiota changes in DR patients because of its low cost, mature protocols, and high sensitivity to low-biomass samples. Although 16S sequencing excels at answering the taxonomic question ‘who is there,’ its resolution is typically limited to the genus level. This makes it difficult to distinguish species or strains accurately, and it cannot directly provide functional gene information (15). This resolution limitation is particularly critical in DR studies, as different strains—even within the same species—can have vastly different metabolic potentials and effects on host immunity.

In contrast, metagenomic shotgun sequencing overcomes these limitations by directly sequencing all genetic material in a sample, providing species-level (and even strain-level) identification along with comprehensive functional gene profiles (70). However, the high cost of metagenomic sequencing, its sensitivity to host DNA contamination, and its substantial demand for computational resources have limited its widespread application in large-scale clinical cohorts. Although 16S sequencing correlates with metagenomic results in overall community structure analysis, significant discrepancies may arise when identifying specific disease-related biomarkers. Thus, careful selection of the technical platform is essential during DR biomarker validation.

### 3.2 Analysis of microbial metabolites

The gut microbiota generates numerous metabolites through its metabolic activities. These compounds enter the host circulation and affect multiple organs, including the retina. Untargeted metabolomic profiling is a comprehensive analysis of small molecule metabolites in biological samples (such as feces and serum) through techniques like liquid chromatography-mass spectrometry (LC-MS) or gas chromatography-mass spectrometry (GC-MS) (71). The metabolic profiles of the gut and serum are altered in DR patients. For instance, the levels of certain SCFAs—including acetate, propionate, and butyrate—may correlate with DR development and progression (72). Targeted metabolomics is a quantitative analysis method focusing on specific pathways or known bioactive metabolites. Studies have shown that ethanolamine alleviates retinal inflammation in retinal explant cultures and diabetic rat models, reduces leukocyte adhesion, and inhibits microglial activation. Ethanolamine may serve as a DR biomarker in well-controlled diabetic patients and is associated with DR progression (73). Metabolomics helps clarify the molecular mechanisms of host–microbiota interactions and reveals how gut microbes affect retinal health through their metabolic products.

### 3.3 Animal model research

Animal models are indispensable for revealing the causal relationship and underlying mechanisms of the gut-retina axis. The streptozotocin (STZ)-induced diabetic mouse/rat model is widely used to study the pathophysiological changes of DR and the effects of gut microbiota intervention. STZ destroys pancreatic β cells, leading to insufficient insulin secretion and thereby inducing the occurrence of diabetes and DR (74). Germ-free or antibiotic-treated mouse models are used to study the impact of microbiota absence on DR development. In these models, researchers transplant DR patient microbiota into germ-free mice through fecal microbiota transplantation (FMT) to observe whether DR-like lesions develop, thereby establishing causality (8).

In DR research, although animal models have played a significant role in elucidating the mechanisms of the gut-retina axis, substantial gaps remain in translating these findings to human applications. 1. Species differences: Rodents differ markedly from humans in gut microbiota composition, physiology, immune responses, and disease pathophysiology. Thus, interventions effective in animals often fail to produce the same results in humans. 2. Disease complexity: DR is a multifactorial, multi-stage disease involving hyperglycemia, inflammation, oxidative stress, neurodegeneration, and microvascular damage (75). 3. Probiotic colonization resistance: Animal experiments are often conducted in germ-free or simplified microbiota conditions, which do not reflect the complex intestinal ecology of humans. 4. Ethical and regulatory Constraints: Human clinical trials require rigorous ethical review and regulatory approval. Compared with animal experiments, human studies face stricter requirements for sample size, trial design, and control groups (76).

Bridging this translational gap requires multifaceted efforts. First, develop animal models that better mimic human DR pathophysiology, or use *in vitro* models such as organoids for initial screening. Second, gain deeper understanding of the precise molecular mechanisms of the gut–retina axis—including how microbial metabolites such as SCFAs influence retinal cells and inflammatory pathways—to enable more targeted interventions. Finally, well-designed, adequately powered, multicenter clinical trials are needed to validate the safety and efficacy of probiotics and fecal microbiota transplantation (FMT) in DR patients (69). Additionally, integrating technologies such as metagenomics and metabolomics to enable personalized analysis of patients’ gut microbiota holds promise for achieving precision medicine in microbial interventions.

### 3.4 Methodological heterogeneity in clinical sample studies

A major challenge in DR gut microbiota research is the significant methodological heterogeneity that complicates direct comparison or integration of findings across studies. First, the standardization of sample collection and processing is insufficient. Fecal samples—the primary source for gut microbiota analysis—are significantly affected by collection timing, storage conditions, and choice of DNA extraction kits. All these factors can greatly influence final sequencing results (77). Moreover, poor control of confounding factors is another major source of methodological heterogeneity. Dietary patterns, age, body mass index (BMI), history of antibiotic use, and comorbidities (such as hypertension and hyperlipidemia) all strongly influence gut microbiota composition (75). Finally, differences in the data analysis process have exacerbated inconsistencies in the results. Given these challenges, future DR microbiome research should focus on establishing a standardized, multicenter collaborative network to unify sample collection, processing, and data analysis procedures. Integrating the large-scale screening advantages of 16S sequencing with the functional analysis capabilities of metagenomic sequencing through a multi-omics approach will be essential for uncovering the molecular mechanisms of the gut–retina axis. Only by overcoming methodological heterogeneity and deepening our understanding of microbial functional mechanisms can the gut microbiome become an effective tool for precise DR diagnosis and treatment.

### 3.5 Applications and limitations of Mendelian randomization studies in the gut-retina axis

Mendelian randomization (MR) is an epidemiological method that uses genetic variants as instrumental variables to infer causal relationships between exposures and outcomes. In gut–retina axis research, MR methods are primarily applied in the following areas: (1) Revealing the causal association between gut microbiota and DR. MR studies utilize genome-wide association study (GWAS) data of the gut microbiome as the exposure and GWAS data of DR as the outcome, aiming to identify potential causal relationships between the gut microbiota and DR. For example, one study employed MR methods to investigate the causal associations between 211 gut microbial taxa and various retinal diseases, including DR. The findings suggested that certain gut microbes—such as unclassified genera within the Ruminococcaceae family, Lactobacillus, and Clostridium—have a causal link with DR development and progression (78). (2) investigating the mediating role of gut metabolites in DR. The gut microbiota influences host physiology through its metabolites. MR studies can be used to assess the causal role of these gut-related metabolites, particularly SCFAs such as propionate, in DR pathogenesis (79).

However, when interpreting MR results, several limitations must be recognized. First, data sources and quality: MR studies typically rely on publicly available GWAS summary data, the quality of which—such as sample size, population homogeneity, and phenotype definition—directly affects the reliability of MR results (80). The heritability of the gut microbiome is relatively low, and its composition and function are heavily influenced by environmental and lifestyle factors. This makes it infeasible to use microbial genetic variation directly as an instrumental variable. Second, horizontal pleiotropy is a core threat to MR analysis. Horizontal pleiotropy occurs when instrumental variables affect the outcome through pathways independent of the exposure, violating a key MR assumption. Third, determining the direction of causality: Although MR aims to address reverse causation, bidirectional MR should still be implemented with caution to avoid potential reversal of causality—that is, the disease status itself may alter exposure levels (81). Fourth, the challenge of multivariable exposure: In the gut-retina axis, multiple microbes and metabolites may jointly influence retinal diseases. Traditional univariate MR struggles to distinguish their individual independent effects (82). Finally, lack of biological validation: MR studies provide statistical causal inference, but their findings still require mechanistic research and experimental verification to establish biological evidence. For example, although MR can indicate a causal relationship between gut microbiota and DR, the specific molecular pathways involved still need further experimental confirmation (8).

In summary, MR is a powerful tool for investigating causal relationships within the gut–retina axis and has shown great potential in retinal disease research. However, researchers must carefully consider the assumptions and limitations of MR studies when designing, conducting, and interpreting studies. Combining MR with other approaches—such as *in vitro* experiments, animal models, or clinical trials—for multi-angle validation is essential to achieve comprehensive and reliable scientific conclusions.

## 4 Potential therapeutic strategies targeting the gut microecology in diabetic retinopathy

### 4.1 Probiotic and prebiotic interventions

Given the key role of gut microbiota in DR pathogenesis, modulating the intestinal flora has emerged as a promising therapeutic strategy. Probiotics and prebiotics are the two main classes of gut microbiota regulators. Probiotics are live microorganisms that, when consumed in sufficient quantities, confer health benefits on the host. They improve metabolic indicators and clinical outcomes in DR patients through multiple mechanisms (83). For example, Bifidobacterium animalis subsp. lactis BB-12 plays a role in maintaining the intestinal barrier and regulating inflammation. Further research has shown that BB-12 reduces obesity, decreases liver steatosis, improves retinal vascular health and vision, and affects the Treg/Th17 balance and intestinal dysbiosis in diabetic mice (84).

Probiotics influence systemic immunity by modulating gut-associated lymphoid tissue (GALT), reducing the production of pro-inflammatory cytokines such as IL-1β, IL-6, and TNF-α, thereby alleviating retinal inflammation. A randomized, double-blind clinical trial in DR patients evaluated the effects of probiotic supplements on metabolic indicators and clinical signs. The results indicated that probiotic supplementation significantly improved insulin metabolism, high-density lipoprotein cholesterol (HDL-C) levels, and some biomarkers of oxidative stress and inflammation (85). Probiotics alleviate retinal damage caused by oxidative stress by generating antioxidant substances or regulating the activity of antioxidant enzymes in the host. They improve insulin sensitivity, lower blood sugar levels, and possibly regulate blood lipids, all of which help slow DR progression (86, 87).

Prebiotics are dietary components not digested or absorbed by the host but that selectively stimulate the growth and activity of beneficial gut bacteria. Human milk oligosaccharides (HMOs) promote the growth of beneficial bacteria such as Bifidobacterium, improving gut microbiota structure. HMOs have demonstrated immunomodulatory effects, enhancing immune function and reducing allergic reactions, which may help alleviate the inflammatory response in DR (88). Prebiotics are fermented by gut bacteria to produce SCFAs, especially butyrate, propionate, and acetate. These metabolic products have multiple benefits for intestinal health and systemic metabolism (47).

The strain-specific nature of probiotics presents challenges in development and application. First, identifying which strains are effective against DR requires extensive screening and mechanistic studies. Currently, clinical research on probiotic interventions for DR is relatively limited, with most studies still at the animal experimental stage. Second, the effectiveness of probiotics may be influenced by factors such as individual gut microbiota composition, diet, and genetic background (89). Therefore, even verified effective strains may exhibit variable effects across individuals. Finally, the storage, transportation, and delivery technologies for probiotics also need further optimization to ensure their activity and efficacy upon reaching the gut.

### 4.2 Targeting microbial metabolites

Metabolic products generated by the gut microbiota, such as SCFAs, influence the inflammatory and oxidative stress status of the retina through the bloodstream, providing potential targets for the treatment of DR. Butyrate supplements improve retinopathy in a DR mouse model, which is related to the regulation of the intestinal microbiota and associated SCFAs. Butyrate exerts protective effects by regulating inflammation and oxidative stress through G protein-coupled receptors (GPCRs) such as GPR41, GPR43 and GPR109A, and by influencing gene expression through histone deacetylase (HDAC) inhibition (47). In the T2DM mouse model, db/db mice with free access to food showed reduced production of tauroursodeoxycholic acid (TUDCA), resulting in low circulating TUDCA levels that failed to bind retinal TGR5 receptors. This led to acellular capillary formation and DR development. In contrast, db/db mice subjected to intermittent fasting exhibit a healthy gut microbiota, increased TUDCA production, and sufficient TUDCA to bind retinal TGR5 receptors, thereby preventing DR (67).

### 4.3 Dietary intervention

Diet is a key factor in shaping the composition, function, and diversity of the gut microbiota. Different dietary patterns profoundly affect the gut microbiota. The Western diet—typically high in saturated fat, sugar, and calories—promotes harmful bacterial growth, reduces beneficial bacteria, increases pro-inflammatory metabolites such as LPS and TMAO, and intensifies systemic inflammation and oxidative stress, thereby accelerating DR development (8, 14). Dietary fiber serves as a substrate for beneficial intestinal bacteria, such as Bifidobacterium and Lactobacillus, which ferment it to produce SCFAs, especially butyrate. Multiple studies have shown that a diet high in fruits, vegetables and fish (such as the Mediterranean diet) may protect against DR development (90). Probiotics are live microorganisms that are beneficial to the health of the host. Prebiotics are selectively fermented dietary components that can promote the growth of beneficial bacteria in the gut. Supplementing probiotics and prebiotics can improve the composition of the intestinal microbiota, enhance intestinal barrier function, and reduce inflammatory responses (88). Docosahexaenoic acid (DHA), an important Omega-3 fatty acid, has been shown to be distributed throughout the zebrafish body and may be beneficial to retinal health. In DR prevention, omega-3 fatty acid supplementation exerts protective effects through anti-inflammatory actions (91).

### 4.4 Fecal microbiota transplantation

FMT involves transferring the fecal microbiota from a healthy donor into a patient’s gastrointestinal tract to restore normal gut microbial structure and function. FMT has been successfully used to treat recurrent infections and has been investigated for obesity, T2DM, inflammatory bowel disease, and some autoimmune and neurological disorders (92, 93). By introducing the diverse microbiota from healthy donors, FMT corrects gut dysbiosis in DR patients, increase beneficial bacteria, and reduce harmful bacteria (94). A healthy gut microbiota enhances the tight junctions between intestinal epithelial cells, reduces intestinal permeability, and thereby decreases the entry of bacterial products and inflammatory factors into the bloodstream. FMT reduces pro-inflammatory cytokine production and increase anti-inflammatory cytokine levels by restoring gut flora balance, thereby alleviating systemic chronic inflammation and oxidative stress (92).

Although FMT application in DR treatment is still in the early exploratory stage, its potential in modulating gut microbiota and improving DR pathophysiology has attracted attention. Overall, FMT is considered safe and well tolerated, even for high-risk patients. Most short-term risks are minor and usually related to delivery methods (95).

FMT standardization challenges include the following: 1. Donor screening: Donor stool quality is critical to FMT success. Although strict donor screening processes are currently in place to exclude potential pathogens and high-risk individuals, screening criteria still vary among institutions. The optimal donor screening protocol continues to evolve, with a need for more comprehensive assessment of donors’ metagenomic and metabolomic profiles to ensure both efficacy and safety (96). 2. Stool suspension preparation and storage: FMT products can be either fresh or frozen fecal microbiota. There is currently no standardized consensus on the best methods for preparing stool suspensions, including concentration, dosage, and storage conditions. While freezing offers convenience, it may affect the viability of certain bacterial strains.

3. Delivery methods: FMT can be administered through various routes, including colonoscopy, nasogastric tube, and oral capsules. Effectiveness and safety may differ by route, but no clear guidelines currently recommend the optimal route for specific diseases (97).

In addition to standardization issues, FMT faces other challenges. First, FMT transplants an entire microbial ecosystem, and its precise mechanism of action is highly complex and difficult to control or predict. This makes FMT application in complex diseases such as DR particularly challenging, given that DR pathophysiology involves multiple interacting pathways. Second, the potential long-term risks of FMT still require further investigation, particularly in immunocompromised patients.

### 4.5 Potential therapeutic agents targeting the gut-retina axis

Growing evidence indicates that various natural active compounds and small molecules exert retinal protective effects indirectly by modulating gut microbiota composition and metabolites. These agents, acting through the “gut-retina axis” mechanism, offer novel drug candidates for DR treatment.

Natural products and their active components are a major focus in this field. Gypenoside A (GPA), a key active compound derived from Gynostemma pentaphyllum, significantly ameliorates retinal microvascular lesions in db/db diabetic mice when administered via ocular surface delivery as a nanoformulation (GPA-loaded mPEG-PLGA nanoparticles). GPA inhibits high-glucose-induced ferroptosis in retinal microvascular endothelial cells by blocking the Nrf2-Keap1 interaction through binding to the Kelch domain of Keap1, thereby activating the Nrf2/HO-1/GPX4 antioxidant pathway (5). The novel polysaccharide THPE2-F derived from Tetrastigma hemsleyanum effectively alleviated retinal damage in STZ-induced DR mouse models by suppressing the ferroptosis pathway through downregulation of the ferritin autophagy receptor NCOA4. This suggests that polysaccharide natural products are promising DR therapeutics (98).

Traditional Chinese medicine (TCM) formulas also show potential in treating DR via the gut-retina axis. Chen’s Jinshui Pills (CJSP) modulate gut microbiota metabolism, increasing the levels of unsaturated fatty acids such as docosahexaenoic acid (DHA) in feces, thereby activating the colonic PPARγ signaling pathway, preserving intestinal barrier integrity, and suppressing gut-derived inflammation (6). By integrating fecal microbiome and metabolomics analyses, this study reveals a complete therapeutic chain: “gut microbiota–metabolites–intestinal barrier–systemic inflammation–retina.” Mudan granules (MuD) identified FBN2 as a key target in STZ-induced diabetic mice through TMT-based quantitative proteomics. They suppressed retinal fibrosis and neovascularization by inhibiting the TNF-α/NF-κB/IL-6 inflammatory pathway and modulating the FBN2/TGF-β1/VEGFA signaling axis (99).

The development of nanomedicine delivery systems has provided key technologies for addressing challenges such as low drug bioavailability and difficulty in crossing the blood-retinal barrier. A mitochondria-targeting DNA tetrahedron nanosystem (PP-TRh-RSV), which loads resveratrol onto rhodamine 19-modified tetrahedral framework nucleic acids (tFNAs) and is coated with PEGylated protamine (PP), enables precise targeting to mitochondria and real-time fluorescent tracking. This nanosystem significantly suppresses retinal neovascularization and retinal degeneration in db/db mice by modulating the RAGE/NF-κB/iNOS and RAGE/NF-κB/VEGF signaling pathways (7). Engineered extracellular vesicles (EVs) specifically deliver interleukin-4 to microglia, enhancing their phagocytic capacity via the GAS6-MERTK pathway, thereby clearing abnormal blood vessels and disrupting the interaction between microglia and pathological vessels. This study demonstrates that EVs serve as a durable, efficient, and safe tool for modulating microglia, offering a potential strategy for restoring immune balance in DR (100).

In summary, potential therapeutic agents targeting the gut-retina axis have advanced from proof-of-concept stages to active compound discovery and nanomedicine development. Future research should focus on: identifying more bioactive compounds capable of modulating the gut microbiota-retina axis through high-throughput screening; elucidating the precise molecular targets of these drugs in both the gut and retina using multi-omics approaches; developing drug delivery systems with excellent bioavailability in ocular tissues to facilitate the clinical translation of these candidate therapeutics (**Table 1**).

**Table 1:** A systematic summary of the gut-retina axis mechanisms, key microbial metabolites, signaling pathways, immune dysregulation, research methodologies, and therapeutic strategies in diabetic retinopathy.

Aspect	Key mechanisms / findings	Supporting evidence	Therapeutic strategies	Future directions
1. Microbiota Dysbiosis in DR	• **Increased Firmicutes/Bacteroidetes ratio** • **Reduced butyrate-producing bacteria** (e.g., Blautia, Faecalibacterium) • **Enriched LPS-producing bacteria** (e.g., Bacteroides, Proteobacteria) • **Altered β-diversity** in DR vs. T2DM vs. controls	16S rRNA sequencing, metagenomics, clinical cohort studies (12–15)	• Probiotics (e.g., Bifidobacterium BB-12) • Prebiotics (e.g., HMOs, fiber) • Fecal microbiota transplantation	• Causality in humans (Mendelian randomization needed) • Personalized microbiota profiling • Long-term stability of interventions
2. Key microbial metabolites				
LPS	• Activates **TLR4/NF-κB pathway** in retina • Induces **microglial M1 polarization** • Disrupts **blood-retinal barrier** via RPE & endothelial injury • Promotes **leukocyte adhesion & capillary** **occlusion**	In vitro models, animal studies, clinical associations (17–25)	• TLR4 antagonists • Barrier enhancers (e.g., butyrate) • Anti-inflammatory agents	• Tissue-specific targeting of TLR4 • LPS as a diagnostic biomarker
TMAO	• **Independent risk factor** for DR severity • Activates **P2X7/NLRP3 inflammasome** in retinal endothelial cells • Promotes **endothelial senescence & oxidative stress**	Plasma metabolomics, network toxicology, molecular docking (26–32)	• Dietary choline/carnitine restriction • TMA lyase inhibitors • NLRP3 inhibitors	• TMAO as therapeutic target • Mechanistic links to VEGF & angiogenesis
Bile Acids	• **TGR5 activation** protects retinal vasculature via RhoA/ROCK inhibition • **FXR modulation** influences inflammation & metabolism • **Altered BA profiles** associated with DR risk	Animal models, serum metabolomics, receptor studies (33–36)	• TGR5 agonists • FXR modulators • Synbiotic formulations (e.g., β-glucan + probiotics)	• BA receptor roles in retinal cells • BA metabolomics in DR progression
SCFAs	• **Butyrate, acetate, propionate** are reduced in DR • **Anti-inflammatory & barrier-protective** effects via GPCRs (GPR41/43/109A) • **Inhibit HDACs**, modulate gene expression	Mouse models, metabolomics, retinal explant cultures (37–42)	• SCFA supplementation • High-fiber diets • Probiotics that enhance SCFA production	• Optimal SCFA dosing & delivery • Retinal SCFA receptor mapping
3. Signaling pathways				
Inflammatory	• **TLR4/NF-κB & IL-6/STAT3** activation by microbial products • **STING pathway** involvement in oxidative stress & inflammation	Cell culture, animal models, pathway inhibitors (43–51)	• NF-κB inhibitors (e.g., albiflorin) • STAT3 modulators • STING pathway blockers	• Crosstalk between pathways • Cell-type-specific signaling
Metabolic	• **AGE/RAGE, polyol, hexosamine** pathways interact with gut-derived signals • **PKC & PI3K/AKT/HIF-1α/VEGF** pathways drive angiogenesis	Genetic & pharmacological models (52–58)	• RAGE antagonists • PKC inhibitors • PI3K/AKT modulators	• Pathway integration in vivo • Dual-target therapies
4. Immune Dysregulation	• **Th17/Treg imbalance** driven by dysbiosis • **Microglial activation & macrophage** **infiltration** • **Increased pro-inflammatory cytokines** (IL-1β, IL-6, TNF-α)	Flow cytometry, cytokine assays, retinal immunohistochemistry (59–61)	• Th17 inhibitors • Treg enhancers • Microglial modulators	• Immune cell trafficking mechanisms • Temporal dynamics in DR progression
5. Research Methodologies	• **16S rRNA & metagenomic sequencing** for microbiota profiling • **Metabolomics** (LC-MS/GC-MS) for metabolite analysis • **Animal models**: STZ-induced, germ-free, FMT-transplanted	Technical validation studies, model comparisons (62–67)	• Multi-omics integration • Humanized mouse models • Organoid & co-culture systems	• Standardization of methods • Translational biomarkers
6. Clinical Translation	• **Probiotics improve metabolic &** **inflammatory markers** in DR patients • **Dietary interventions** (Mediterranean, high-fiber) show protective effects • **FMT & synbiotics** show promise in preclinical models	Randomized controlled trials, cohort studies, meta-analyses (68–78)	• Personalized nutrition plans • Combination therapies (gut + eye) • AI-guided treatment optimization	• Large-scale RCTs needed • Safety & long-term efficacy • Regulatory pathways for microbiota-based therapies

This table integrates preclinical and clinical evidence, highlights current therapeutic approaches, and identifies critical research gaps for future investigation. DR, diabetic retinopathy; LPS, lipopolysaccharide; TMAO, trimethylamine N-oxide; SCFAs, short-chain fatty acids; FMT, fecal microbiota transplantation; RCT, randomized controlled trial.

## 5 Conclusion

The emerging paradigm of the gut-retina axis fundamentally reshapes our understanding of DR pathogenesis. It shifts the perspective from a localized ocular vascular disorder to a systemic condition intricately linked to intestinal homeostasis. Compelling evidence indicates that gut microbiota dysbiosis plays a pivotal role in DR progression. This dysbiosis compromises intestinal barrier integrity, facilitating the translocation of microbial products into systemic circulation. These metabolites, alongside dysregulated bile acids and diminished SCFAs, subsequently drive retinal pathology through multiple interconnected mechanisms: perpetuating chronic low-grade inflammation, exacerbating oxidative stress, disrupting neurovascular unit integrity, and promoting aberrant angiogenesis.

Although the gut–retina axis concept has opened new avenues for understanding and treating DR, several challenges remain. Further mechanistic studies are needed to clarify how the gut microbiota precisely affects retinal health through its metabolic products, immune regulation, and inflammatory pathways. For instance, Mendelian randomization studies have been used to explore the causal relationship between the gut microbiota and DR (16). The composition of the intestinal microbiota varies greatly among different individuals. Therefore, future treatment plans must account for individual patient characteristics and develop precise intervention strategies. Currently, most studies remain at the stage of animal models. More high-quality clinical trials need to be carried out to evaluate the efficacy and safety of interventions based on the gut-retina axis in DR patients.

High-throughput sequencing, metagenomic sequencing, and metabolomics analysis will facilitate a more comprehensive understanding of gut microbiota and metabolite changes in DR patients. These technologies will provide a basis for identifying new biomarkers and therapeutic targets. Artificial intelligence (AI) and machine learning have demonstrated great potential in DR diagnosis and management. In the future, AI could analyze complex gut microbiome data, identify specific microbial features related to DR, and guide the formulation of personalized treatment plans.

In conclusion, the gut-retina axis offers new perspectives for the prevention and treatment of DR. By elucidating the interaction mechanisms between the gut microbiota and the retina and developing targeted intervention strategies, we can expect to bring more effective and safer treatment options to DR patients.
